# Using Motor Imagery to Study the Neural Substrates of Dynamic Balance

**DOI:** 10.1371/journal.pone.0091183

**Published:** 2014-03-24

**Authors:** Murielle Ursulla Ferraye, Bettina Debû, Lieke Heil, Mark Carpenter, Bastiaan Roelof Bloem, Ivan Toni

**Affiliations:** 1 Donders Institute for Brain, Cognition and Behaviour, Centre for Cognitive Neuroimaging, Radboud University Nijmegen, Nijmegen, the Netherlands; 2 Grenoble Institut des Neurosciences, INSERM U838, Université de Grenoble, Grenoble, France; 3 School of Kinesiology, University of British Columbia, Vancouver, Canada; 4 Department of Neurology, Donders Institute for Brain, Cognition and Behaviour, Radboud University Nijmegen Medical Center, Nijmegen, the Netherlands; University Medical Center Groningen UMCG, Netherlands

## Abstract

This study examines the cerebral structures involved in dynamic balance using a motor imagery (MI) protocol. We recorded cerebral activity with functional magnetic resonance imaging while subjects imagined swaying on a balance board along the sagittal plane to point a laser at target pairs of different sizes (small, large). We used a matched visual imagery (VI) control task and recorded imagery durations during scanning. MI and VI durations were differentially influenced by the sway accuracy requirement, indicating that MI of balance is sensitive to the increased motor control necessary to point at a smaller target. Compared to VI, MI of dynamic balance recruited additional cortical and subcortical portions of the motor system, including frontal cortex, basal ganglia, cerebellum and mesencephalic locomotor region, the latter showing increased effective connectivity with the supplementary motor area. The regions involved in MI of dynamic balance were spatially distinct but contiguous to those involved in MI of gait (Bakker et al., 2008; Snijders et al., 2011; Crémers et al., 2012), in a pattern consistent with existing somatotopic maps of the trunk (for balance) and legs (for gait). These findings validate a novel, quantitative approach for studying the neural control of balance in humans. This approach extends previous reports on MI of static stance (Jahn et al., 2004, 2008), and opens the way for studying gait and balance impairments in patients with neurodegenerative disorders.

## Introduction

Neuroimaging has been used extensively to study the neurophysiology of human motor control, focusing mainly on hand, arm, and foot movements [1], [2], [3], [4]. Studying the cerebral bases of whole body movements in humans has proven technically less tractable, and this has hampered the acquisition of knowledge about the mechanisms of gait and balance control. Neuroimaging methods that allow for whole-body movements have either superficial cortical coverage, as is the case with near-infrared spectroscopy [5] or limited temporal resolution as is the case with single-photon emission computed tomography or positron emission tomography [6], [7]. Recently, new opportunities for understanding the cerebral control of human whole-body movements have emerged from the combination of quantitative motor imagery protocols and fMRI. This approach quantifies cerebral activity while subjects imagine a particular movement. This approach has been successful in studying static balance control and gait in humans [8], [9], [10], [11], [12], [13]. The use of motor imagery is particularly relevant for studying the cerebral correlates of balance because it leads to the selection of balance-related motor programs, without the confounds of performance-related variation in somatosensory reafference. Thus, motor imagery makes it possible to distinguish feedforward control of balance from changes in somatosensory feedback during balance performance. This issue becomes particularly important for extending the approach to clinical populations, e.g. Parkinson's disease patients, where interactions between motor execution and sensory reafference from balance failures can be prominent. Besides specificity for planning-related components of balance control, motor imagery offers the possibility to study the selection of balance-related motor plans in a recumbent position, i.e. in a position compatible with techniques like fMRI. This is important for exploiting the high spatial resolution and whole-brain coverage afforded by those techniques. However, using motor imagery also requires objective quantification of first-person motor imagery during the scanning period. We developed an experimental protocol to investigate *dynamic* balance control.

Surprisingly, indeed, *dynamic* balance control has not yet been investigated, although it is a major function of the postural control system. Instability should be thought of as context-specific, where each individual is at risk of falling in different contexts [14], [15]. The postural control system includes a number of components underlying the ability to stand, walk and interact safely with the environment. Understanding those components requires examining their individual neural activation patterns under controlled task conditions. Accordingly, we explore the relation between cerebral circuits supporting dynamic postural challenges (this study) and circuits supporting upright quiet stance [10], [11] and gait [8]. Furthermore, given the clinical relevance of understanding failures of balance control, and given that those failures often occur while moving, this study aimed at designing a motor imagery protocol that would enable one to isolate the cerebral circuits supporting *dynamic* postural challenges.

In contrast to the limited knowledge available on the neural control of dynamic balance in humans, several studies have investigated postural control in quadrupedal animal models, revealing how portions of the brainstem, the pons, and the mesencephalic locomotor region (MLR, including the pedunculopontine and cuneiform nuclei) control postural muscle tone and coordinate stepping movements along the midsaggital plane [16], [17], [18]. In macaques, these regions receive projections from the supplementary motor area (SMA) [19], [20], and inhibition of the SMA disturbs postural control [21]. Similar cortico-pontine projections have been recently described in humans [22], but there is no evidence for their role in balance control, either through direct long-range connections or via thalamic mediation [23].

Here, we designed a behaviorally controlled motor imagery protocol to isolate cerebral activity and connectivity involved in dynamic balance control. Furthermore, we explored the spatial relation between cerebral circuits supporting dynamic balance, static balance and gait, by comparing our results with those of previous studies on MI of stance and gait [8], [12]. By moving backward and forward along the midsaggital plane, participants standing on a balance board aimed a laser dot (mounted on the balance board) at targets of different sizes, placed at different distances. In order to control for subjects engagement in motor imagery during fMRI, we exploited the fact that both physical performance and motor imagery of a given action are influenced by task difficulty according to Fitt's law [24], [25]. Accordingly, the designed postural aiming task manipulated the difficulty of dynamic balance by varying the extent and accuracy of oscillations on a trial-by-trial basis. Afterwards, the participants imagined performing the same dynamic balance task while their BOLD-fMRI responses and leg electromyography were recorded. They pressed a button to indicate the onset and offset of their imagery. Cerebral responses specific to dynamic balance control were isolated by comparison with a visual imagery task involving the same sensory input and motor output (index finger flexions resulting in button presses), but in which participants did not imagine swaying voluntarily.

## Materials and Methods

### Subjects

Twenty healthy right-handed subjects (10 men; age 20.2±1.8 years, mean ± SD) took part in the study after giving written informed consent according to the declaration of Helsinki. All subjects had normal or corrected-to-normal vision, and no neurological or orthopaedic disturbances. The study was approved by the local ethics committee, *Commissie Mensgebonden Onderzoek regio Arnhem, Nijmegen* (CMO2001/095). The ethical approval of the abovementioned research protocol is valid until 12/31/2013.

### Experimental settings

**Figure 1:**
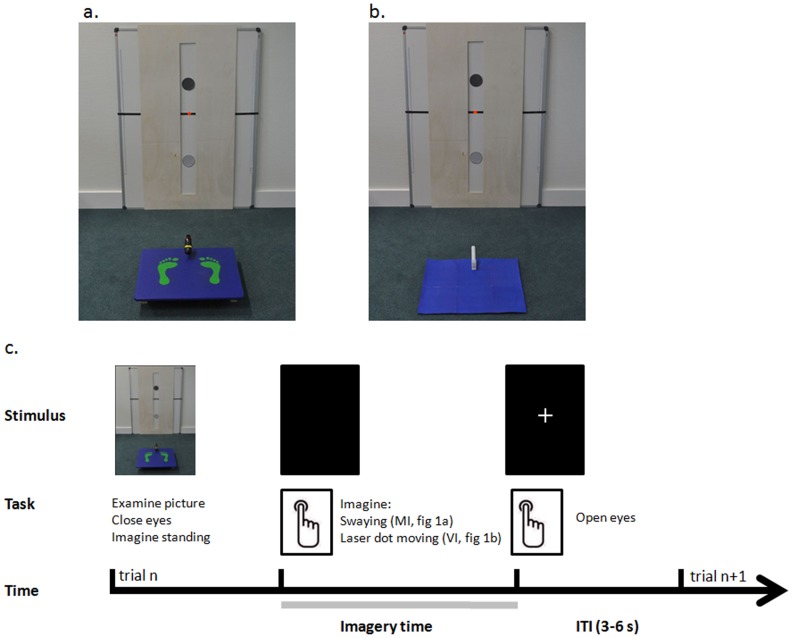
Experimental setup. Examples of photographs of the setup presented to the subjects during the a. motor imagery (MI), and b. visual imagery (VI) tasks. Both photographs show a whiteboard with circle targets in the middle and a black line with the red dot from the laser (highlighted here) in between. During MI trials, a balance board is present in front of the whiteboard. During VI trials, a blue carpet replaces the balance board. In these examples, the trajectory length is long (80% of subjects' maximal sway) and the target size is large (8 cm of diameter); c. Time course of motor imagery trials. During each trial, after a short inspection of the photo on display, the subjects closed their eyes and imagined standing on the balance board. The subjects were asked to press a button with the index finger of their right hand to signal that they had started imagining swaying on the balance board. The subjects were asked to press the button again when they imagined the balance board was back to the starting position, after having aimed the laser dot at both targets. Following the second button press, a fixation cross was presented on the screen and the subjects could open their eyes. The duration of the inter-trial interval (ITI) was 3–6 s.

The subjects first physically performed a dynamic balance task (DBT), followed by imagery of this very same task in the MR scanner. The DBT used a forward/backward swaying balance board, with a laser pointer secured to the front (Figure 1a). The pointer projected a red laser dot on a 90×120 cm whiteboard located at a distance of 1.5 meter in front of the subject. When subjects were in their resting balance position on the board, the laser would point at a given position on the whiteboard, in between the two targets. This position was set as the starting position. When subjects actively swayed forward or backward, the red laser dot would move downward or upward on the board, respectively. Pairs of circular targets of two different diameters (8 cm: large target size; 2 cm: small target size) were vertically aligned on the whiteboard. The large target allowed subjects to sway with fewer constraints when reaching the target. The small target forced subjects to sway carefully to land and remain within the target area. To normalize task difficulty between participants, the distance between the two targets of a pair was individually adjusted for each subject, based on the maximal forward and backward sways that each subject was able to perform without losing balance.

### Experimental design

The difficulty of the task was manipulated *via* three factors: sway amplitude, target size and initial direction of sway. Regarding sway amplitude, two distances were selected: 80% (large amplitude sway) and 60% (small amplitude sway) of the subject's maximal forward and backward sway, measured at the onset of the experiment. The average distance between the targets across subjects was 61.4 cm [range: 41.0–73.0 cm] for the large amplitude sway condition and 46.1 cm [range: 30.5–54.5 cm] for the small amplitude sway condition. To manipulate initial direction of sway, each target pair included a dark and a light grey target (first and second target to aim at, respectively). Combination of these three factors (two sway amplitudes, two target sizes and two initial directions of sway) yielded a total of eight different conditions.

### Experimental procedure

There were two experimental sessions, performed on a single day. In the first session, the subjects were administered the Revised Version of the Vividness of Motor Imagery Questionnaire (VMIQ-2, 10 min) [26] and physically performed the Dynamic Balance task (45 min). In the second session, the subjects performed two imagery tasks in counterbalanced order across subjects. In one of the imagery tasks, the subjects had to perform motor imagery of the very same dynamic balance task they just physically performed. The other imagery task was a visual imagery control task.

### VMIQ-2

This questionnaire screens subjects' ability to perform motor imagery. The subjects are required to rate the vividness of their imagery on a 5-point Likert scale for a set of 12 actions. Three different imagery perspectives are considered, including kinaesthetic imagery, our perspective of interest. In this imagery perspective, subjects are instructed to imagine themselves performing a given action from ‘within’, that is, trying to mentally perceive the associated sensations and muscle contractions [27]. This perspective recruits the neural networks involved in programming the actual actions. Motor imagery can otherwise be “external”, referring to self-visualisation of a movement from either the first person (ie, seeing the movement from one's own point of view) or the third person (ie, seeing oneself carrying out the task from outside) perspective (see Maillet et al., 2012 for a review on motor imagery of gait). The subjects of this study were informed about these different imagery perspectives, and their score on the VMIQ was collected. They were specifically requested to adopt the kinaesthetic perspective to perform motor imagery of balance. The cut-off score for the inclusion of individuals in imagery studies is 36 for each perspective (from 12: “perfectly clear and as vivid as normal feel of movement” to 60: “no sensation at all, you only know that you are thinking of the skill”) [26]. The subjects had a mean score of 24.4±7.1 and no one had a score greater than 36. Therefore, all the 20 subjects were considered able to perform motor imagery using the kinaesthetic perspective and were included in the second part of the study.

### Dynamic balance task

The subject stood barefoot on the balance board, with feet parallel at shoulder width, and a stopwatch in the right hand. The subject was asked to sway forward or backward on the board and aim the red laser dot at the darker target first, then at the lighter target, and then return the red laser dot to its starting position. Each trial therefore consisted of a sequence of three sway directions, the order of which differed as a function of the initial direction of sway: Forward-Backward-Forward or Backward-Forward-Backward. Subjects pressed a button to mark the beginning and the end of the movement to allow recording of the trial duration.

The subject was asked to actively sway forward or backward while keeping the knees and trunk extended, heels and toes on the board, and arms at the sides. The subject's body had to remain orthogonal to the balance board, and not to the floor in order for a trial to be valid. The laser beam had to remain within the perimeter of each target for about 1 second before the subject could move to the next target. Trials with obvious knee or trunk flexion, or lack of proper stabilization of the laser beam within the target were excluded and repeated. Before the start of the measurements, subjects were given a few practice trials until they were able to comply with these requirements (≈10 min). Trials for each experimental condition were repeated three times, with a pseudo-randomized order, for a total of 24 trials (8 conditions ×3 repetitions).

### Motor imagery task; visual imagery task

Subjects performed the imagery tasks while lying supine in the MR scanner, starting with a few practice trials to make sure they understood and followed the instructions before onset of the MRI measurements. Head movements were minimized by an adjustable padded head holder. Each trial started with the presentation of a photograph of one of the experimental conditions experienced during the DBT (Figure 1). The photograph was projected onto a screen at the back of the scanner and was seen through a mirror above the subjects' heads. Stimuli presentation was controlled through a PC using the Presentation software (Neurobehavioural systems, Albany, USA).

During motor imagery (MI) trials, the photograph showed the balance board used during the DBT (Figure 1a). Subjects were asked to imagine swaying on the board (using first-person kinaesthetic perspective) to aim at the targets, as was done during the DBT, including sway corrections due to over and undershooting of the targets. During visual imagery (VI) trials, the photograph showed a blue carpet instead of the balance board (Figure 1b). Subjects were asked to imagine seeing the laser dot smoothly moving at a constant speed from the middle of one target to the middle of the other target, without overshooting the targets. A video showing the red laser dot autonomously moving on the whiteboard was first shown to the subjects in order for them to have an experience on which to ground visual imagery, and to provide them with background knowledge comparable to that existing for MI (in which subjects experience the balance board before engaging in the MI task). The video of the red laser dot illustrated the type of motion (i.e. a smooth velocity profile), and each subject was only shown the video of a single trial so as not to influence their imagery duration.

A trial started with displaying a photograph that subjects could inspect as long as needed. Subjects then closed their eyes and pressed a button to mark the onset of their imagery period. Motor responses (i.e. index finger flexions resulting in button presses) were recorded via a button box positioned on the right of the subject's abdomen. Subjects pressed the button again (and opened their eyes) when they imagined either reaching the initial position of the balance board (MI trials) or seeing the laser dot aiming at that position (VI trials). The difference between the two subsequent button presses represented the imagery duration. After the second button press, a fixation cross was presented on the screen until the onset of the next trial (inter-trial interval, ITI, 3 to 6 seconds). The MI and VI tasks were performed in two experimental sessions, separated by a break outside the scanner. For each imagery task, subjects performed 80 trials, in pseudo-randomized order across the eight experimental conditions. The two experimental sessions were matched for length after MR acquisition, considering the first 15–20 minutes of performance for each task.

### Data collection

MR images were acquired on a 3 T Trio MRI system (Siemens, Erlangen, Germany), using a 32 channel head coil. A multi-echo sequence with four echoes (TE: 9.4, 21.2, 33, 45 ms, TR: 2410 ms) was used to improve signal to noise and reduce inhomogeneities [28], with 37 transversal slices; ascending acquisition; voxel size 3.5×3.5×3.0 mm^3^; FOV = 224 mm^2^). High-resolution anatomical images were acquired using an MPRAGE sequence (TR/TE 2300/3.92 ms, 192 sagittal slices, voxel size 1.0×1.0×1.0 mm^3^, FOV 256 mm^2^). The Brainamp ExG amplifier was used to collect muscle activity to control for overt leg movements during task performance in the MR-scanner (MI and VI). Silver/silver-chloride electrodes were placed on the right tibialis anterior in a belly tendon montage. The right patella was used as reference electrode. Following amplification and A/D conversion (Brain Products GmBH, Gilching, Germany), an optical cable fed the EMG signal to a dedicated PC outside the MR room for further off-line analysis (sampling rate: 5000 Hz). MR artefact correction followed the method previously described [29], [30], including low-pass filtering (400 Hz), and down-sampling (1000 Hz). Finally, we applied high-pass filtering (10 Hz, to remove possible movement artefacts), and rectification.

Eye closure/opening were measured during task performance in the MR-scanner with a video-based infrared eyetracker (Sensomotoric Instruments, Berlin, Germany). Movements of the left eye were sampled at 50 Hz and fed to a dedicated PC outside the MR room. Eye closure/opening at imagery onset/offset respectively was visually inspected online to make sure that the subjects followed the instructions to perform the imagery task.

### Behavioural analysis and statistical inference

For each trial, we measured the time between the two button presses that marked the start and the end of the DBT, imagined visual, or balance movements (trial duration). We considered four experimental factors: TASK (three levels: DB, MI, VI); SWAY AMPLITUDE (two levels: Short, Long); TARGET SIZE (two levels: Small, Large); INITIAL DIRECTION OF SWAY (two levels: Forward, Backward). The significance of the experimental factors was tested within the framework of the General Linear Model using a 3×2×2×2 repeated measures ANOVA. When interactions were significant, the origin of the interaction was investigated using planned comparisons. Specifically, we hypothesized to find an effect of amplitude sway across the three tasks, but an effect of target size (reflecting increased control of balance accuracy) only for the DB and MI tasks. The alpha-level of all behavioural analyses was set at p<0.05.

In addition, to ascertain MI of dynamic balance during scanning, we examined whether the trial duration obtained in each task conformed to Fitts' law (Fitts, 1954): Trial duration  =  a + b log_2_ (2*SWAY AMPLITUDE/TARGET SIZE). In the equation, *a* and *b* are constants. The term log_2_ (2*SWAY AMPLITUDE/TARGET SIZE) is called the index of difficulty (ID). It describes the difficulty of the motor tasks. We calculated ID for each of our 4 experimental conditions [i.e., target size (2 levels) and sway amplitude (2 levels)]. Fitts' Law states that trial duration increases linearly with increasing ID. We examined how well trial durations conformed to Fitts' Law by calculating the linear regression of trial duration over ID for each TASK (3 levels, DB, MI and VI) and for each subject separately. Finally, we examined whether the degree to which trial durations conformed to Fitts' Law was different for the different tasks, by considering the effect of TASK (DB, MI and VI) on the variance in trial duration that could be explained by ID (r2) after log transformation, using a repeated measures ANOVA.

### EMG analysis and statistical inference

For each trial of the imagery experiment, we considered the root mean square (rms) of the pre-processed EMG signals measured during the imagery epoch and during the ITI. For each subject, the average rms values of the EMG measured during the imagery epoch was normalized to the average rms values of the ITI epoch on a subject by subject basis, testing for an effect of TASK (MI, VI) with a paired sample t-test. We also tested whether for both MI and VI, the EMG activity differed from the resting condition by comparing the EMG activity evoked during motor imagery and visual imagery with the EMG activity evoked during the inter-trial intervals, using a paired sample t-test on the average root mean square values of the EMG.

### fMRI analysis — pre-processing

Functional data were pre-processed and analyzed with SPM8 (Statistical Parametric Mapping, www.fil.ion.ucl.ac.uk/spm). The first 30 volumes of each participant's data set were discarded to allow for T1 equilibration and calculation of the weighting factor for the multi echo sequence [28]. Afterwards, the image time series were spatially realigned using a sinc interpolation algorithm that estimates rigid body transformations (translations, rotations) by minimizing head-movements between each image and the reference image [31]. Subsequently, the time-series for each voxel was temporally realigned to the acquisition of the first slice. Images were normalized to a standard EPI template centred in MNI (Montreal Neurological Institute) space [32] and resampled at an isotropic voxel size of 2 mm. The normalized images were smoothed with an isotropic 8 mm full-width-at-half-maximum Gaussian kernel. Anatomical images were spatially coregistered to the mean of the functional images [32] spatially normalized by using the same transformation matrix applied to the functional images and finally segmented into grey matter, white matter, CSF and other non-brain partitions [32].

### fMRI analysis — statistical inference (first level)

The ensuing pre-processed fMRI time series were analyzed on a subject-by-subject basis using an event-related approach in the context of the General Linear Model [31]. We considered two models. The first model was aimed at finding regions in which the cerebral response changed as a function of TASK (MI, VI), TARGET SIZE (Large, Small), and SWAY AMPLITUDE (Long, Short), which gave rise to a model with eight different regressors of interest. The model also included a separate regressor of no interest, modelling BOLD activity evoked by button presses, separately for each session. Each effect was modelled on a trial by trial basis as a concatenation of square-wave functions convolved with a canonical haemodynamic response function, down sampled at each scan, generating a total of 10 task-related regressors. For the regressors of interest, onsets of the square-wave functions were time-locked to the button press marking the onset of imagery, and durations corresponded to the imagery time of each separate trial (e.g. time between the two button presses). For the button press regressor, onsets were time locked to the button press marking the onset of imagery, and duration was set to zero. The potential confounding effects of residual head movement-related effects were modelled using the time series of the estimated head movements during scanning. We included the original time series, the squared, the first-order derivatives of the originals and the first-order derivatives of the squared [33]. Data were high-pass filtered (cutoff, 128 s) to remove low-frequency confounds such as scanner drifts. The statistical significance of the estimated evoked hemodynamic responses was assessed using t-statistics in the context of the General Linear Model. For each subject, we calculated contrasts of the parameter estimates for the effects of TASK (i.e. MI, VI), TASK and TARGET SIZE (i.e. MI-Small, MI-Large, VI-Small, VI-Large) and TASK and SWAY AMPLITUDE (i.e. MI-Short, MI-Long, VI-Short, VI-Long). As the behavioural analysis revealed a main effect of Task, correlations between behavioural and neural effect size were assessed by investigating Pearson correlations with alpha level set at p<.05.

### fMRI analysis — statistical inference (second level)

The group-level random effects analysis modelled the experimental variance described by the contrasts involving the TASK and TARGET SIZE factors for each subject by means of a one-way between-subjects analysis of variance (ANOVA), with non-sphericity correction. First, we considered the main effect of TASK (MI, VI). This refers to differential cerebral activity between the two tasks (MI>VI; VI>MI). Second, we looked for MI-specific effects of target size, namely differential cerebral activity evoked during MI between the large and small targets (MI-Small>MI-Large; MI-Large>MI-Small), as compared to the corresponding trials during visual imagery (i.e. a TASK X TARGET SIZE interaction). SPMs of the t statistic for the effects corresponding to these contrasts were created. We report the results of a random effects analysis, with inferences drawn at the voxel level, corrected for multiple comparisons across the whole brain using family-wise error (FWE) correction (p<0.05).

### Region of interest analysis

Besides whole brain analyses, statistical inference was also performed on one particular region of interest: the mid-mesencephalon. In a previous study by our group [12], we showed that this region (coordinates: [0 −28 −20]) which includes the MLR (including the pedunculopontine nucleus and the cuneiform nucleus) showed increased activity during MI of gait in PD patients with freezing of gait. A recent study also showed activation of the same region (local maxima coordinates: [−2 −30 −20]) during MI of gait in healthy subjects [9]. We therefore considered these local maxima and drew a spherical region of interest centred at these coordinates ([0 −28 −20] and [−2 −30 −20]) with a radius of 10 mm. Statistical inference was performed at the voxel-level, with a family-wise error correction for multiple comparisons over the search volume (p<0.05).

### Effective connectivity analysis

After having identified the left thalamus and the MLR as being involved in MI of balance, we performed a post-hoc analysis to explore which brain areas increased their correlation with these two regions during MI of balance. The thalamus was chosen in particular because it is an important relay in the subcortico-cortical motor loop, it plays a central role in sensorimotor integration and is specifically involved in balance control. Therefore, MI of balance was expected to modulate the coupling of the thalamus with the pallidum, the SMA and the MLR. Likewise, based on several connectivity studies [22], [34], [35], [36], [37], we tested the hypotheses that MI of balance modulates the interregional coupling of the right MLR with the thalamus, the SMA and the cerebellum.

During MI, those brain regions could operate independently, or they could increase their correlation as compared to periods of visual imagery. To test for these task-related modulations of inter-regional connectivity, we used the psychophysiological interaction (PPI) method described by Friston et al., [38]. PPI analysis makes inferences about regionally specific responses caused by the interaction between the psychological factor (in this case, performing the MI or the VI task) and the physiological activity in a specified source region. Following functional anatomical considerations (see above), we considered two source regions: the thalamus and the MLR. We also considered a series of target regions: pallidum, MLR, and SMA when considering the thalamus as a source region; and SMA, thalamus, and cerebellum when considering the MLR as a source region.

Each source area was described by a BOLD timeseries that was the first eigenvariate of the timeseries of all voxels within a 6 mm radius sphere centred on the regional local maximum of the SPM{t} relative to MI versus VI in each of the two source regions. First, a PPI analysis for each source region was performed on each subject. Then, for each source region, PPI contrast images of each subject were entered into a one-sample paired t-test at the second level. In addition to a whole brain analysis, we assessed significant voxel-level effects (FWE corrected for multiple comparisons, p<0.05) within the different regions hypothesized to show increased coupling with each of the two source regions, using a search volume defined by those voxels that were activated in the contrast MI>VI (as defined above).

### fMRI analysis — anatomical inference

Anatomical details of significant signal changes were obtained by superimposing the statistical parametric maps on the anatomical sections of a representative subject of the MNI series. When applicable, Brodmann Areas were assigned on the basis of the SPM Anatomy Toolbox [39], i.e., the anatomical position of our significant clusters and local maxima was formally tested against published three dimensional probabilistic cytoarchitectonic maps. Finally, we compared our results to those of a previous study on MI of gait [8], to assess the relative anatomical segregation of the MLR for gait and balance functions. More specifically, we compared the anatomical locations of the cerebral activity found for balance to that of cerebral areas showing increased activity during MI of gait in healthy subjects, as previously reported [8].

## Results

### Behavioural performance

**Figure 2:**
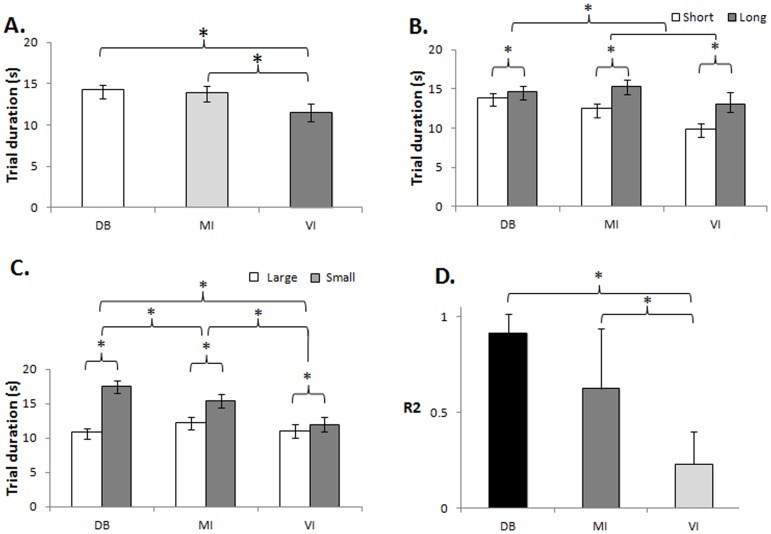
Behavioural results. Trial durations across tasks and conditions. **A**. Trial duration measured in each task [dynamic balance (DB), motor imagery (MI) and visual imagery (VI)]; **B**. Trial duration measured for each trajectory lengths within each task [short (60% of subjects' maximal sway) and long (80% of subjects' maximal sway)]; **C**. Trial duration measured for each target size within each task [large (8 cm) and small (2 cm)], Data represent mean ± SEM; **D**. Average r2 of the correlation between trial duration and ID for each of the different tasks (post hoc comparison of r2 across the different tasks). Data represent mean ± SD; * indicates p<0.05.

Trial durations were significantly shorter for the VI task than for the MI and DB tasks [main effect of TASK: F(2,38) = 3.32, P<0.05; Figure 2A]. Trial durations increased with increasing sway amplitude [main effect of SWAY AMPLITUDE: F(1,19) = 62.73, P<0.001] (Figure 2B) and with decreasing target size [main effect of TARGET SIZE: F(1,19) = 103.73, P<0.001]. There was a significant TASK * SWAY AMPLITUDE interaction: F(2,38) = 5.71, P<0.01. Planned comparisons revealed that the difference in trial durations between the small and large sway amplitude was significant for all three tasks [DBT: F(1,19) = 5.70, p<0.027; MI: F(1.19) = 79.5, p<0.001; VI: F(1.19) = 33.37, p<0.001]. Crucially, the effect of target size was different for the different tasks [TASK * TARGET SIZE interaction: F(2,38) = 45.81, P<0.001; Figure 2C]. Planned comparisons revealed that the increase in trial duration with smaller target size was larger during DB than VI [F(1,19) = 113.19, P<0.001], during MI than VI [F(1,19) = 9.40, P<0.01], and during DB than MI [F1,19) = 41.18, P<0.001]. As predicted from Fitts' law (Fitts, 1954), trial durations correlated linearly with ID for the DB (r^2^ = 0.9±0.1) and MI (r^2^ = 0.6±0.3) tasks but not for VI (r^2^ = 0.2±0.2). Likewise, the variance in trial duration that could be explained by ID (r^2^) was different for the different tasks [main effect of TASK: F(2.38) = 10.1, P = 0.00031, Figure 2D]. The r^2^ was greater for MI than for VI (P = 0.002), and for DB than for VI (P = 0.000), but the r2 did not differ between MI and DB (P = 0.27).

Finally, trial durations were longer in the forward than in the backward initial direction of sway (main effect of INITIAL DIRECTION OF SWAY, F(1,19) = 9.11, P<0.01) although this effect was not modulated by any other factor.

There was similar EMG activity during the two imagery tasks (t(1,17) = 0.83, p = 0.42). In addition, there were no statistically significant differences between the average rms values of the EMG evoked during the imagery epochs and the rest epochs, both for MI (p = 0.97) and for VI (p = 0.89). Hence, differences in actual movements related to overt leg movements during MI of balance did not account for changes in differential (MI compared with VI) cerebral activity.

### Cerebral activity — task effects

**Figure 3:**
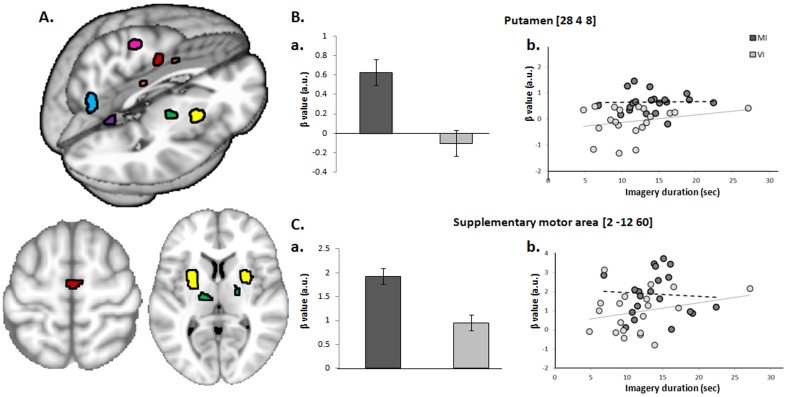
Brain regions activated during motor imagery of dynamic balance. A. Cerebral activity during motor imagery of balance. Statistical parametric map (SPM) of increased activity in the supplementary motor area (red), the dorsal premotor cortex (pink), the middle cingulate cortex (orange), the superior parietal lobule (cyan), the thalamus (green), the putamen (yellow) and the cerebellum (purple), during MI compared to VI [corrected for multiple comparisons (p<0.05) using family wise error (FWE)], superimposed on a 3D rendered brain (upper part) and on axial sections (lower part). B. Putamen activity; a. β values as a function of Task; b. Relationship between behavioral (imagery durations) and neural (β values) changes as a function of Task. Subject-by-subject variance in imagery durations was not correlated with the neural Task-related effects for both the MI (putamen: r = 0.025, p = 0.917) and VI tasks (putamen: r = 0.248, p = 0.291). C. Supplementary motor area activity; a. β values as a function of Task; b. Relationship between behavioral (imagery durations) and neural (β values) changes as a function of Task. Subject-by-subject variance in imagery durations was not correlated with the neural Task-related effects for both the MI (SMA: r = -0.062, p = 0.793) and VI tasks (SMA: r = 0.254, p = 0.279).

We first identified cerebral regions showing significant differential activation during MI compared to VI (Figure 3, Table 1). BOLD signal increased in the right medial frontal cortex and left precentral gyrus. Both clusters were within BA6 [39], with the right medial frontal cortex cluster falling within the SMA-proper and the left precentral cluster falling in the caudal part of the dorsal premotor cortex [40] A third cluster was located in the middle cingulate cortex (CMA), with its local maximum in the right rostral cingulate zone posterior (RCZp) [1], [41]. Significant effects were also found in the left insula, in the left superior parietal lobule (7M, 7P, 7A), in the left thalamus (ventral lateral nucleus, with a trend for its contralateral equivalent, p = 0.078), in the putamen (bilaterally), and in the left globus pallidus. Finally, the cerebellar vermis (culmen, lobule V bilaterally and right tonsil, lobule VIII) also showed increased neural activity during MI as compared to VI.

**Table 1:** Stereotactic coordinates of the local maxima activated in the contrast “MI>VI”.

Search volume	Anatomical label	Funct. label	Hemi.	t-value	p-value	x	y	z
Whole brain	Medial frontal gyrus (BA6)	SMA	R	5.87	0.006	2	−12	60
	Precentral gyrus (BA6)	PMd	L	5.85	0.006	−18	−22	68
	Insula		L	5.52	0.017	−28	20	−20
	Superior Parietal Lobule (7M, 7P, 7A)		L	6.19	0.002	−4	−72	38
	Middle cingulate cortex	RCZp	R	5.98	0.004	8	6	38
	Middle cingulate cortex	RCZp	L	5.62	0.013	−8	8	40
	Thalamus (ventral lateral nucleus)		R	5.02	0.078	14	−16	4
	Thalamus (ventral lateral nucleus)		L	5.89	0.005	−16	−14	10
	Putamen		R	7.58	0.000	28	4	8
	Putamen		L	7.67	0.000	−24	2	14
	Lateral Globus Pallidus		L	6.5	0.001	−20	−6	10
	Cerebellum (Lobule V, Culmen)		R	5.21	0.045	14	−42	−18
	Cerebellum Lobule V, Culmen)		L	6.32	0.001	−4	−54	−2
	Cerebellar tonsil (Lobule VIIIb)		R	5.52	0.017	22	−42	−50
Region of interest	Mesencephalon	MLR	R	4.83	0.002	6	−24	−16
	Pons		R	4.49	0.002	2	−22	−16

Results are corrected for multiple comparisons across the whole brain (FWE<0.05). Stereotactic coordinates are reported in MNI (Montreal Neurological Institute) space. Details on the anatomical and functional labeling can be found in the Results section. L = left; R = right; Funct = Functional.

We performed a further analysis on the putamen and the SMA specifically involved in MI, to test whether there was a direct relationship between behavioral (imagery durations) and neural (β values) changes as a function of Task. We exploited the fact that different subjects of our group showed differences in imagery durations. Figure 3 shows that subject-by-subject variance in imagery durations was not correlated with the neural Task-related effects for both MI (putamen: r = 0.025, p = 0.917; SMA: r = −0.062, p = 0.793) and VI tasks (putamen: r = 0.248, p = 0.291; SMA: r = 0.254, p = 0.279).

**Figure 4:**
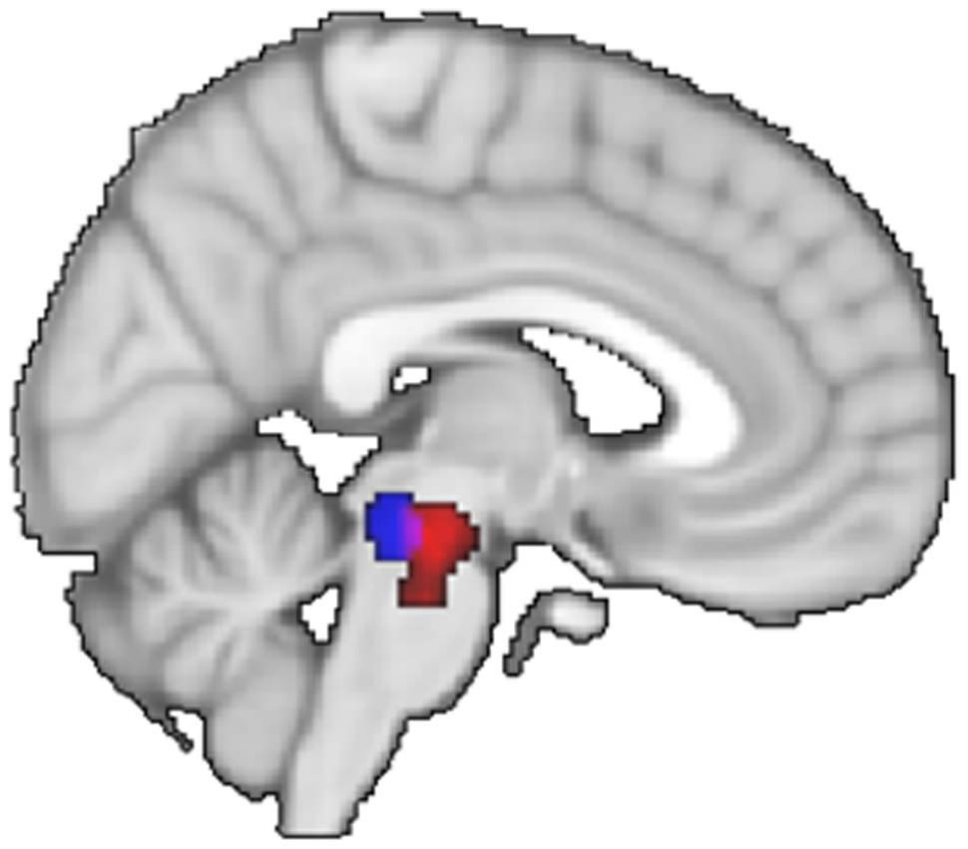
Comparison of gait and dynamic balance related activations in the mesencephalon. Statistical parametric map (SPM) of differential activity between motor imagery of gait (blue) [9], [12] and motor imagery of balance (red).

Setting a ROI on a portion of the brainstem recently found using fMRI to be involved in the neural control of gait in humans [9], [12] revealed increased activity during MI of balance. The local maximum is located slightly anterior, lateral and rostral (6 −24 −16) to previously reported gait-related effects found in PD patients with freezing of gait (0 −28 −20, [12]) and in healthy subjects during MI of gait (−2 −30 −20, [9]) (Figure 4). Differential activity between MI and VI was also found in the pons (2 −22 −16). A post-hoc test for the presence of effects within the location previously found during MI of static balance (−12 −14 −14, [11]) did not reveal any significant effect, suggesting that a different part of the MLR might be involved during dynamic balance.

There were no supra-threshold cerebral differences driven by differences in target size or initial direction of sway during MI compared to VI.

### Cerebral activity — effective connectivity

The results described above indicate that the left thalamus and the right antero-rostral part of the MLR were involved in MI of balance. We used PPI analyses to examine whether their activity influenced the cerebral regions supporting the MI process. There were no significant whole-brain effects, but the volume of interest analysis showed that the left thalamus increased its coupling with the left lateral globus pallidus ([−18 −6 2]; t = 3.95, p = 0.025) as a function of TASK (MI>VI); similarly, the right MLR increased its coupling with the right SMA during MI of balance ([8 −6 62]; t = 3.90, p = 0.032). The connectivity between the thalamus and the MLR ([8 −18 −12], t = 2.95; p = 0.123) was not significantly modulated by the motor imagery task.

## Discussion

This study investigated the neural substrates of dynamic balance using a new experimental protocol that combined fMRI with controlled performance of motor imagery (MI). We characterized cerebral regions sensitive to motoric effects related to dynamic balance control, over and above generic imagery-related effects. Imagery of dynamic balance control recruited cortical and subcortical portions of the motor system, including frontal and parietal cortices, basal ganglia and cerebellum. After pointing out some methodological issues, we discuss the significance of the behavioural data, then examine the brain regions specifically involved in dynamic balance control, and explore their relation to brain regions showing quiet stance and gait-related effects.

### Methodological considerations

There are several methodological issues that should be considered when trying to generalize the findings of the current study. First, the postural incongruency between the imagined movement and the actual position of the subject in the scanner might have increased the cognitive demands of the task and is therefore a clear limitation to the present study. Second, The dynamic balance task used in this study is focused on a particular aspect of active balance control in the mid-sagittal plane, and we do not address possible interactions with other elements of balance control. However, it should be emphasized that body oscillations in the mid-sagittal plane are particularly relevant for clinical practice as, for instance, most of the falls in Parkinson's disease occur for movements along that plane, and especially in the backwards direction [42]. A third issue relates to the interpretation of the results from the spatial comparison between dynamic balance and gait control. Indeed, absence of a gait imagery condition in the design of this study precludes directly contrasting imagery of gait and imagery of balance within the same subjects. These results therefore need to be replicated intra-subjects.

### Behavioural performance

This study was designed to isolate imagery-related effects driven by motoric constraints of dynamic balance, rather than by visual imagery alone or by somatosensory re-afference (that is inherent to actual task performance). The behavioural data recorded during the imagery tasks indicate that the participants were effectively engaged in the imagery tasks, performing different imagery activities under the MI vs. VI conditions. Indeed, imagery times, as actual DB times, increased when participants performed trials with larger sway amplitude and smaller target size. Furthermore, the increase in trial duration with smaller target size was significantly greater during MI and actual performance of the DBT task than during the corresponding visual imagery task. Finally, both DB and MI trial duration was significantly correlated with the motoric index of difficulty of the task. Such correlation did not hold true regarding VI durations. This effect was centrally generated, since EMG activity was well matched across the two imagery tasks. Thus, these behavioural recordings confirm that the current task captures motoric processes also involved in the actual performance of the balance task, and different from basic phenomena shared with the visual imagery task (e.g. processing of the visual stimuli, production of the button presses). It therefore validates our dynamic balance protocol, providing a control for the mental activity deployed by the participants during scanning.

### A distributed cerebral circuit for dynamic balance control

Several portions of the motor system were differentially active during motor imagery of dynamic balance (Table 1). These effects were independent from differences in imagery durations between MI and VI imagery. Cortically, both medial and lateral portions of the frontal cortex were significantly activated (SMA-proper, dorsal premotor cortex), as were a portion of the cingulate motor areas (RCZp), the left insula and the left superior parietal lobule (7M, 7P, 7A). Subcortically, we found differential balance-related effects in the ventrolateral part of the thalamus and in the putamen (bilaterally), in the left globus pallidus, in the cerebellar vermis (culmen, lobule V bilaterally and right tonsil, lobule VIII), in the right anterior part of the MLR, and in the right medial pons. These findings fit with observations from human clinico-pathological reports, and imaging studies of postural control contrasting standing with lying [10], [11], as discussed hereafter. For instance, parietal, frontal and thalamic regions were activated during both dynamic balance (present study) and quiet stance [10], [11]. Diffuse brain MRI abnormalities including white matter hyperintensities [43] and gray matter atrophy [44] have been reported in the frontal and parietal cortices of individuals with balance impairments. The present findings extend the known role of frontal and parietal cortex in visual guidance and control of limb movements [45], [46] to axial balancing movements, including a role in the integration of somatosensory signals into representation of body parts and the guidance of movements in space.

The importance of the thalamus for maintaining upright posture is supported by clinical reports of body tilts and falls following ischemic infarction of its posterolateral part [23]. Barra et al. [47] found that the sense of verticality required the integrity of the posterolateral thalamus encroaching mostly on the ventro-posterior lateral nuclei. Finally, reduced activation of the thalamus was reported in a fMRI study of MI of standing in patients with progressive supranuclear palsy with prominent postural imbalance and falls. In these patients, higher sway values and frequency of falls were also associated with decreased regional glucose metabolism in the thalamus [48]. Our finding of bilateral, although asymmetrical, activation in the ventral lateral part of the thalamus concur with these observations, further confirming the role of the ventral part of the thalamus in postural control.

The clinical relevance of balance centers in the brainstem is supported by the description of a patient who was unable to stand (astasia) following an hemorrhage into the tegmentum of the posterior midbrain involving the right pedunculopontine area [49]. Further evidence for a role of the MLR in modulating posture and gait in humans rests on post-mortem studies of cholinergic cell groups showing (a) a cholinergic neuronal loss in the pedunculopontine of patients with advanced Parkinson's disease or with progressive supranuclear palsy [50] in whom gait disorders, postural instability and falls are dominant from the onset of the disease, and (b) that the extent of the degeneration of the pedunculopontine neurons correlates with the patients' levels of premortem axial dysfunction [51]. In addition, improvements of postural stability following deep brain stimulation of the pedunculopontine nucleus area have been reported [52], suggesting possible functionally specific subterritories for gait and balance within the MLR, as previously reported in the animal [53].

Compared to MI of quiet stance, a few differences likely inherent to the dynamic aspect of our balance task are worth mentioning. First, we observed a strong activation of the basal ganglia, which was not consistently reported during quiet standing [10]. The basal ganglia could be involved in the release of automatic postural responses to balance threats, or to the subjects' anticipation of postural instability while approaching the stability limits. Some neurons in the putamen have been shown to discharge in apparent anticipation of predictable events (Mink, 1996), a finding that has been extended to balance control using a task involving recognition of unstable postures in a dynamic context [54].

In addition, while the SMA was inconsistently activated during MI of quiet standing, it showed a strong response during MI of dynamic balance, possibly reflecting the coordination of postural adjustments to the voluntary sway movements.

Lastly, while Jahn et al. (2008) reported activation in the cerebellar hemispheres, the dynamic balance task used in this study seemed to preferentially recruit the median cerebellum. Both extensive research on balance and clinical pathological studies have clearly demonstrated that the cerebellum plays a central role in postural control [55], [56]. Its median part, the vermis, was shown to be particularly involved in the active maintenance of body balance [55], while its lateral part could be less specifically linked to motor control [54].

### Relation between dynamic balance and gait control

The motor imagery effects captured in the current study for motor imagery of dynamic balance and in Bakker et al [8] for motor imagery of gait are spatially contiguous. This is true both at the cortical and subcortical levels, lending further support to the idea of functionally specific subterritories in various brain regions, or reflecting their somatotopic organization. Assuming that the trunk is the primary motor effector for balance, and the legs are the primary motor effectors for gait, then the relative positions of the frontal effects reported in Bakker's and our studies fit with previously reported somatotopic maps of the trunk and the legs [57], [58], [59]. The trunk is represented more laterally than the legs on the precentral gyrus [58], [59], and more rostrally in the SMA [57].

The effects observed in medial parietal and cingulate cortex are congruent with a recent report of gray matter changes in these regions following improved performance on a dynamic balance task [60]. They also fit with a few sparse reports on trunk-related activity in these regions [61], although previous work has largely focused on orofacial, forelimbs and hindlimbs representations, omitting the trunk. Subcortically, we found differential balance-related effects in the ventrolateral part of the thalamus and in the putamen (bilaterally), in the left globus pallidus, in the cerebellar vermis (culmen, lobule V bilaterally and right tonsil, lobule VIII), in the right anterior part of the MLR, and in the right medial pons. The latter effect is congruent with the idea of a topographical organization of the pons and of the MLR. In the pons, the trunk would be represented medially, as reported in physiological mapping of the primary motor cortex projections to the pons in the monkey [62], and the hindlimbs laterally [63]. In the MLR, we report a gradient between a medio-caudal part involved in gait [9], [12] and a latero-rostral part involved in balance (Figure 4). It remains to be seen whether this spatial segregation is function-specific, related to different control parameters between balance and gait (postural muscle synergies versus interlimb coordination respectively), or different rhythms (low versus fast respectively), or effector specific, related to different effectors (trunk versus legs respectively). We speculate that the overlap between these two functional poles could reflect coordinated leg and trunk movements evoked during both gait and balance control [64], in line with the known functional organization of this region [65], [66]. Deciphering the organization of the MLR at this level of functional resolution in healthy subjects advances our knowledge of this structure, and it might improve targeted stimulation of the pedunculopontine nucleus in patients with Parkinson's disease [52], [67],[68]. However, it remains to be seen whether the spatial relation of the cerebral substrates of gait and balance, observed here at the group-level and through qualitative comparisons across studies, can be confirmed within individual subjects and within the same study.

### Mesencephalic locomotor region (MLR) - supplementary motor area (SMA) connectivity

The MLR is directly connected to the supplementary motor area in humans [22], [34], [36], [69]. Here we show a dynamic aspect in the connectivity of these two structures, namely increased connectivity between MLR and SMA when the imagery content involves balance control.

We failed to observe task-related modulations of connectivity between the MLR and the thalamus, although there are anatomical connexions between these structures [34], [35], [36]. Instead, increased functional connectivity was found between the thalamus and the globus pallidus. Overall, although our data cannot resolve the anatomical direction of these connectivity changes, we suggest that the supplementary motor area, the MLR, the thalamus and the basal ganglia are part of a network working interactively to control dynamic balance.

## Conclusions

This study builds on the approach used by Bakker et al. [70] for mapping cerebral structures involved in human gait control, extending it to the control of dynamic balance. Our results confirmed the involvement of cerebral regions previously identified in human clinico-pathological reports and in imaging studies on postural control. Fronto-parietal regions, basal ganglia and medial cerebellum are involved during MI of dynamic balance. We also provide some evidence for at least partly spatially segregated cerebral circuits supporting gait and dynamic balance control. The approach used in this study opens the way for directly studying the pathophysiology underlying dynamic balance impairments in older individuals, and in patients with motor disorders.
